# Commentary: Improvements in Cycling Time Trial Performance Are Not Sustained Following the Acute Provision of Challenging and Deceptive Feedback

**DOI:** 10.3389/fphys.2017.00031

**Published:** 2017-01-25

**Authors:** Ren-Jay Shei, Emily M. Adamic, John S. Raglin, Kevin G. Thompson, Timothy D. Mickleborough

**Affiliations:** ^1^Department of Medicine, University of Alabama at BirminghamBirmingham, AL, USA; ^2^Department of Kinesiology, School of Public Health-Bloomington, Indiana UniversityBloomington, Indiana, USA; ^3^Faculty of Health, Research Institute for Sport and Exercise, University of CanberraCanberra, ACT, Australia

**Keywords:** pacing strategy, competition, deception, power output, feedback

We read with interest the recent study by Jones et al. (2016) who found performance gains elicited by deceptive feedback were not sustained in a subsequent ride-alone time trial (TT). Although they employed a design somewhat similar to a study from our laboratory (Shei et al., 2016), we feel the discrepancies between their findings and ours are the result of several methodological differences.

First, the final trial of our study followed a deceptive feedback trial, and it is possible the inclusion of an avatar on the computer screen contributed in part to the enhanced performance observed in this trial by an implicit competition effect. The presence of an avatar could be perceived as either competition or support (e.g., domestiques in mass-start cycling competitions or pacers in running races). To date, studies investigating the performance influence of a second athlete have been equivocal (Bath et al., 2012; Corbett et al., 2012). Presently, little is known regarding how cyclists perceive an avatar, warranting further investigation to advance our understanding of how competition is perceived. In our study the performance improvement (i.e., completion time) of 2.1% with- and following deceptive feedback was similar to the 1.7% improvement found by Stone et al. (2012) using deceptive feedback (individual responses given in Figure 1). Stone et al. (2012) also reported the presence of an accurate (no deception) avatar elicited only a 1.0% performance improvement, significantly (*P* < 0.05) less than the improvement observed with deceptive feedback. Hence, we propose the presence of an avatar alone is only partially responsible for the magnitude of the performance improvement observed in our study.

**Figure 1:**
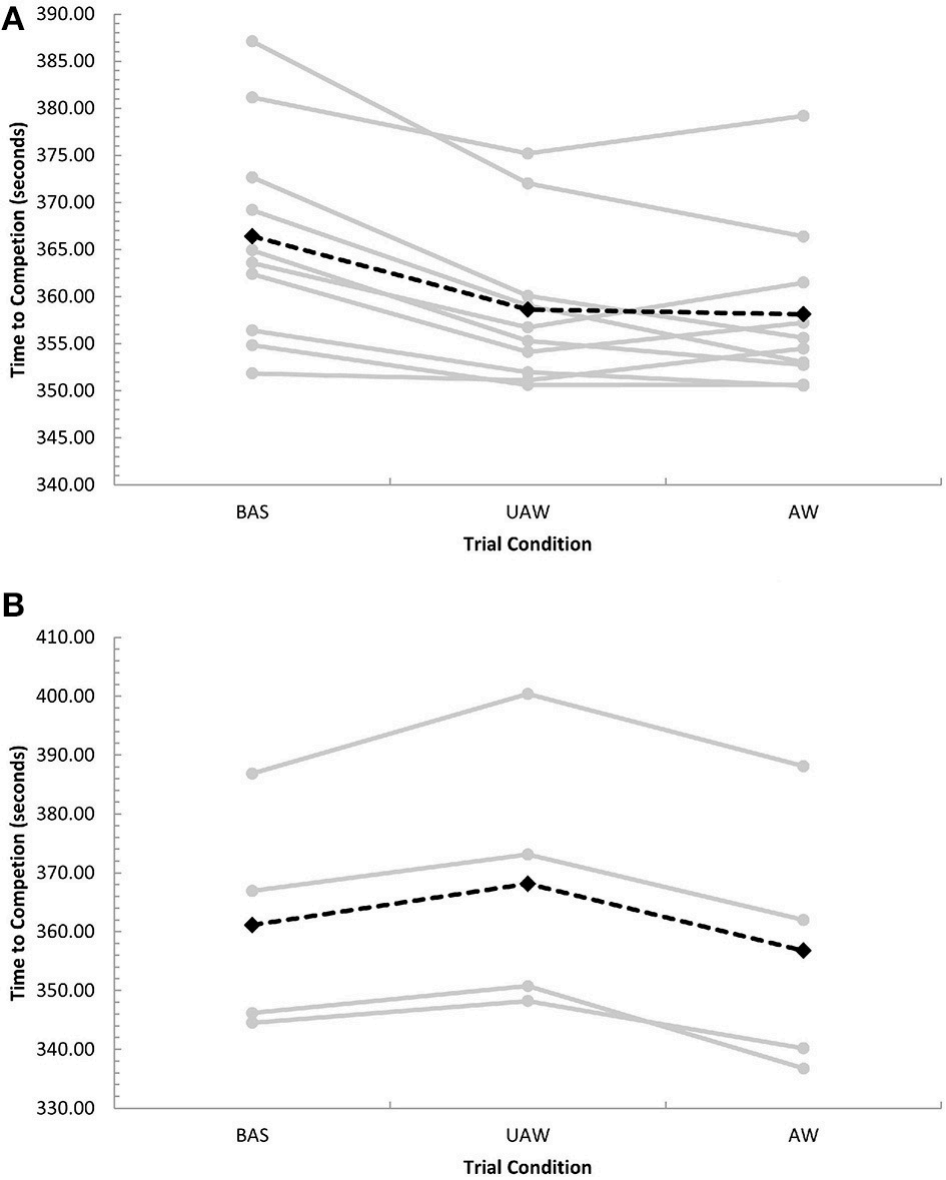
**(A)** Individual (gray, closed circles) and mean (black, closed diamonds) completion times for subjects who improved in the UAW condition compared to BAS (*n* = 10). **(B)** Individual (gray, closed circles) and mean (black, closed diamonds) completion times for subjects who did not improve in the UAW condition compared to BAS (*n* = 4). BAS, baseline trial (ride-alone); UAW, unaware trial (ridden with on-screen avatar set at 102% of baseline mean power output, unbeknownst to subjects, who were falsely informed that the avatar represented their baseline performance); AW, aware trial (ridden with subjects informed that the on-screen avatar was set at 102% of baseline mean power output and informed of the true nature of the previous UAW trial). For full description of conditions, (see Shei et al., 2016).

Second, Jones et al. (2016) manipulated *speed* by 2%, whereas we increased *power* by 2%. The relationship between speed and power is not linear; modeling indicates a 1% increase in speed equates to a ~2.9% increase in mean power output (Flyger, 2008). Therefore, the deceptive feedback manipulation in Jones et al. (2016) was over twice the magnitude (2% in speed, or roughly 5.8% in power) of our 2% power increment. Jones et al. (2016) reported a 17 second improvement in performance time, equivalent to approximately a 1% improvement in completion time. The completion time improvement compares closely to the avatar-alone effect observed by Stone et al. (2012), but is smaller than the improvements associated with deceptive feedback found in our study (+2.1%) and by Stone et al. (2012) (+1.7%). Additionally, the performance improvement from the fastest baseline trial to the pacer trial reported by Jones et al. (2016) did not differ between the accurate and deceptive feedback groups. This, in tandem with the smaller performance improvement of 1%, suggests the performance improvement observed in the pacer trial could well have been due to the mere presence of the pacer in the deceptive feedback manipulation (i.e., PACER trial), rather than the deceptive manipulation. The 2% increase in speed (~5.8% increase in power output) may have exceeded the functional increment which can elicit a further improvement in performance. Other work which implemented a deceptive 5% increment in speed and power respectively above baseline failed to yield any improvement in performance in 20 km (Micklewright et al., 2010) and 4 km (Stone, 2012) TTs. The magnitude of the deceptive feedback manipulation in these studies might have been so great as to not escape detection by participants, who then adopted a conservative pacing strategy.

Finally, overall performance time or mean power output does not imply the use of identical pacing strategies across trials. Indeed, subjects can achieve identical mean power outputs despite purposefully different pacing strategies, which can impact fatigue development and performance (Atkinson and Brunskill, 2000). While Jones et al. (2016) did not observe differences in mean power output or performance time in the subsequent trial compared to baseline, their data appear to indicate an increased rate of the rise in power output during the last 4 km of the subsequent trial in both the deceived and accurate feedback groups (Figure 3, Jones et al., 2016) despite similar mean power outputs at 12 and 16 km. The participants may have been able to voluntarily increase their power output at some point over the last 4 km compared to their baseline trial, and analysis of the serial distribution of power output could clarify whether changes in pacing occurred between trials.

Changes in the serial distribution of power output could be due to the “end-spurt” phenomenon, an increase in power output and anaerobic energy contribution in the later stages of an event. The end-spurt has been found to occur reliably in the last 10% (or 400 m) of a 4 km time trial (Stone et al., 2011, 2012), and was observed in our study during all trials. In addition, end-spurts have been observed in TTs over 20 km and 30 mins (Thomas et al., 2012; Abbiss et al., 2016), so it is likely an end-spurt would occur during the last 10% (1600 m) of a 16.1 km time trial, although the onset and duration of an end-spurt likely differs based on distance, adopted pacing strategy, and other factors (Abbiss and Laursen, 2008). Importantly, even small increases in the end-spurt, can result in a meaningful increase in overall performance, despite not being reflected in mean power output over the last 4 km (Atkinson and Brunskill, 2000). We feel it remains plausible the “end-spurt” can be enhanced to a meaningful extent through an appropriate level of negative-deception feedback (via an ~2% change in mean power output in 4 km cycling TTs) by eliciting a greater contribution from previously inaccessible anaerobic energy reserves (Hettinga et al., 2006; Stone et al., 2011, 2012).

In summary, differentiating between the gain in performance from competition alone, and the additive gain from deception is important. Differences in cycling speed, pacing strategy, and time trial distance can each influence energy reserves, the magnitude of the “end-spurt,” and overall performance, ultimately determining an individual's response to deception. The variable of deception remains a useful tool in pacing research and it may ultimately become a viable training tool for improving cycling performance.

## Author contributions

R-JS, EA, JR, KT, and TM all contributed substantially to the conception or design of the work, the drafting the work and revising it critically for important intellectual content, and the final approval of the version to be published. All authors agree to be accountable for all aspects of the work in ensuring that questions related to the accuracy or integrity of any part of the work are appropriately investigated and resolved.

### Conflict of interest statement

The authors declare that the research was conducted in the absence of any commercial or financial relationships that could be construed as a potential conflict of interest.

## References

[B1] Abbiss C. R. Laursen P. B. (2008). Describing and understanding pacing strategies during athletic competition. Sports Med. 38, 239–252. 10.2165/00007256-200838030-00004 18278984

[B2] Abbiss C. R. Thompson K. G. Lipski M. Meyer T. Skorski S. (2016). Pacing differs between time- and distance-based time trials in trained cyclists. Int. J. Sports Physiol. Perform. 11, 1018–1023. 10.1123/ijspp.2015-0613 26868360

[B3] Atkinson G. Brunskill A. (2000). Pacing strategies during a cycling time trial with simulated headwinds and tailwinds. Ergonomics 43, 1449–1460. 10.1080/001401300750003899 11083127

[B4] Bath D. Turner L. A. Bosch A. N. Tucker R. Lambert E. V. Thompson K. G.. (2012). The effect of a second runner on pacing strategy and RPE during a running time trial. Int. J. Sports Physiol. Phys. Perform. 7, 26–32. 10.1123/ijspp.7.1.26 21941007

[B5] Corbett J. Barwood M. J. Ouzounoglou A. Thelwell R. Dicks M. (2012). Influence of competition on performance and pacing during cycling exercise. Med. Sci. Sports Exerc. 44, 509–515. 10.1249/MSS.0b013e31823378b1 21900846

[B6] Flyger N. (2008). Variability in competitive performance of elite track cyclists. ISN Bull. 1, 27–32.

[B7] Hettinga F. J. De Koning J. J. Broersen F. T. Van Geffen P. Foster C. (2006). Pacing strategy and the occurance of fatigue in 4000-m cycling time trials. Med. Sci. Sports Exerc. 38, 1484–1491. 10.1249/01.mss.0000228956.75344.91 16888463

[B8] Jones H. S. Williams E. L. Marchant D. Sparks S. A. Bridge C. A. Midgley A. W.. (2016). Improvements in cycling time trial performance are not sustained following the acute provision of challenging and deceptive feedback. Front. Physiol. 7:399. 10.3389/fphys.2016.00399 27713701 PMC5031686

[B9] Micklewright D. Papadopoulou E. Swart J. Noakes T. (2010). Previous experience influences pacing during 20 km time trial cycling. Br. J. Sports Med. 44, 952–960. 10.1136/bjsm.2009.057315 19364755

[B10] Shei R.-J. Thompson K. Chapman R. Raglin J. Mickleborough T. (2016). Using deception to establish a reproducible improvement in 4-km cycling time trial performance. Int. J. Sports Med. 37, 341–346. 10.1055/s-0035-1565139 26855435

[B11] Stone M. R. (2012). Effects of Deception on Exercise Performance: Implications for Determinants of Fatigue in Humans. PhD Dissertation, Northumbria University, Newcastle-upon-Tyne. 10.1249/MSS.0b013e318232cf77 21886012

[B12] Stone M. R. Thomas K. Wilkinson M. Jones A. M. St Clair Gibson A. Thompson K. G. (2012). Effects of deception on exercise performance: implications for determinants of fatigue in humans. Med. Sci. Sports Exerc. 44, 534–541. 10.1249/MSS.0b013e318232cf77 21886012

[B13] Stone M. R. Thomas K. Wilkinson M. St Clair Gibson A. Thompson K. G. (2011). Consistency of perceptual and metabolic responses to a laboratory-based simulated 4,000-m cycling time trial. Eur. J. Appl. Physiol. 111, 1807–1813. 10.1007/s00421-010-1818-7 21222130

[B14] Thomas K. Stone M. R. Thompson K. G. St Clair Gibson A. Ansley L. (2012). Reproducibility of pacing strategy during simulated 20-km cycling time trials in well-trained cyclists. Eur. J. Appl. Physiol. 112, 223–229. 10.1007/s00421-011-1974-4 21533808

